# CCR4 and CCR5 Involvement in Monocyte-Derived Macrophage Migration in Neuroinflammation

**DOI:** 10.3389/fimmu.2022.876033

**Published:** 2022-05-12

**Authors:** Jong Youl Kim, Jiwon Kim, Meiying Huang, Renée Kosonen, Jong Eun Lee

**Affiliations:** ^1^Department of Anatomy, Yonsei University College of Medicine, Seoul, South Korea; ^2^Graduate School of Medical Science, Brain Korea 21 Project, Yonsei University College of Medicine, Seoul, South Korea; ^3^Brain Research Institute, Yonsei University College of Medicine, Seoul, South Korea

**Keywords:** neuroinflammation, microglia, monocyte-derived macrophage, CCR4, CCR5

## Abstract

Microglia, resident macrophages in the brain, play major roles in neuroinflammation after an acute many neurological diseases, including stroke. Our recent animal stroke model showed that interleukin (IL)-4 and IL-13 released by microglia are converted into monocyte-derived macrophages. However, the correlation with the migration mechanism of these cells is still unclear. This study aimed to clarify the effect of these cells on their migration and to identify potential targets that influence neuroinflammatory conditions. Inflammatory conditions were induced by lipopolysaccharide (LPS) treatment in *in vitro* and *in vivo* models. Cell migration was observed using transwell assay, and target chemokines were screened using the proteome profiler array in the *in vitro* model. Intravital, IVIS, and CLARITY imaging were used in the *in vivo* model. After LPS (1 ng/ml) treatment in BV2 (microglia cell line) and J774 (monocyte/macrophage cell line) cells, BV2 migration was approximately two-fold more enhanced compared to J774 migration. Overall, six types of chemokine C-C motif ligands (CCLs) were detected from the BV2 conditioned medium with LPS. These CCLs were related to C-C motif receptor (CCR)4 and CCR5. In the *in vivo* model, CCR4 and CCR5 antagonist significantly inhibited the migration of monocyte-derived macrophages to brain tissue following LPS (5 µg) treatment. In conclusion, the chemokines released by microglia may influence migration of monocyte-derived macrophages in necroinflammation conditions inducted by microglial activation. CCR4 and CCR5 expressed on monocyte-derived macrophages interacted with these chemokines and induced migration. Therefore, CCR4 and CCR5 may be explored as new therapeutic targets for neuroinflammation.

## Introduction

Post-stroke inflammation leads to worse neurological outcomes by secondary injury (1). The recruitment of both microglia and immune cells in brain parenchymal tissue after ischemic stroke can accelerate brain infarction and increase the infarct volume (2).

Microglia, resident macrophages in the brain, play a key role in neuroinflammation after ischemic stroke (3, 4). Microglia activation is known to induce various inflammatory and cytotoxic mediators that contribute to cell damage and death, leading to exacerbated brain injury (5, 6). Microglia activation by brain injury has been shown to lead to the migration and proliferation of immune cells and the release of various inflammation-related effectors, including superoxide, nitric oxide, proteases, and cytokines (7). This is accompanied by phagocytosis of damaged cells (8). Importantly, some of these responses may exacerbate brain injury and thus provide potential therapeutic targets. Ischemic damage and an altered blood-brain barrier (BBB) induce recruitment of circulating immune cells (9). Recruitment of monocyte-derived macrophages to the ischemic brain is coordinated by inflammatory cytokines, such as adhesion molecules and chemokines (10). The infiltrating monocytes can transform into macrophages; these infiltrating monocytes differentiate into two phenotypes: pro-inflammatory macrophages (M1) and anti-inflammatory macrophage (M2) (11). Activated macrophages in the ischemic area may control microglial polarization by regulating excessive pro-inflammatory response of M1 and driving protective polarization of M2 (12). Brain tissue damage by microglia and macrophage activation is directly associated with neuronal cell death (13, 14).

Our recent study demonstrated that pro-inflammatory monocytes migrate shortly after an ischemic stroke and are converted by interleukin (IL)-4 and IL-13 into anti-inflammatory macrophages that attenuate ischemic stroke (15). However, potential therapeutic targets that regulate the recruitment of monocyte-derived macrophage in neuroinflammation have not been investigated. Further, evidence on the association between monocyte-derived macrophage and microglia is rare. Thus, this study aimed to identify the influencing factors of monocyte-derived macrophage migration in neuroinflammation and potential therapeutic targets.

## Materials and Methods

### Cell Culture and Animals

Mouse monocyte cell line (J774) was obtained from Korean Cell Line Bank and cultured in RPMI 1640 medium (Hyclone, UT, USA) supplemented with 2 mM L-glutamine, 10% fetal bovine serum (FBS, Hyclone), and 1% penicillin-streptomycin (Hyclone). Murine microglia cell line (BV2) was obtained from Ajou University College of Medicine, Chronic Inflammatory Disease Research Center. BV2 cells were cultured in RPMI 1640 medium (Hyclone) supplemented with 10% FBS (Hyclone) and 1% penicillin-streptomycin (Hyclone). Both cells were kept in a 37°C incubator supplemented with 5% CO_2_ and sub-cultured in 90 × 20 mm cell culture dishes (2-3 days after seeding at a density of 1 × 10^6^ cells/ml) when confluence was >80%.

For the animals, healthy male C57BL/6 mice (weight, 25-30 g; age, 8-12 weeks; Central Lab Animal Inc) and CCR2:: red fluorescent protein (RFP) (weight, 25-30 g; 8-12 weeks; Jackson Lab) mice were prepared for *in vivo* experiments. A total of 50 mice were used in this study. All animal experiments were performed as per the guidelines of The Institutional Animal Care and Use Committee of Yonsei University Health System and according to the National Institutes of Health guidelines.

### Transwell and Chemokine Assays

J774 or BV2 were placed in the lower compartment of a six-well chamber with poly-l-ornithine-coated cover slides attached to the base, and transwell coated with 3-µm microporous membranes was inserted in the upper compartment of the same chamber (Costar). J774 and BV2 (2 × 10^6^ cells/ml) were cross-seeded in the upper compartment with conditioned medium (CM) and CM with lipopolysaccharide (LPS; CML) from BV2 and J774, respectively. After a 48-hour reaction, cells that accumulated in the lower compartment were subjected to Hoechst staining (33342, Thermo). The remaining cells in the upper compartment were stained with crystal violet (Sigma). All slides were then viewed under an optical microscope (Eclipse Ti2; Nikon) and a confocal microscope (LSM 700; Carl Zeiss).

To screen the CM and CML of BV2, a proteome profiler assay was carried out using the Mouse Chemokine Array Panel A (R&D system) according to the manufacturer’s instructions. The blots containing captured chemokines in the membrane were oxidized with the ECL™ Western Blotting Analysis System (GE Healthcare) and visualized using the LAS 4000 mini (Fujifilm). Finally, the chemokines were quantified using HLImage++ (Western Vision software) for array analysis.

### Immunocytochemistry and Immunoblotting

To confirm the receptor of J774, cells were fixed with 4% paraformaldehyde (PFA) for 15 minutes and blocked with 3% bovine serum albumin for 1 hour. They were then treated with mouse anti-CCR4 (1:1000; Santa Cruz Biotechnology) and rabbit anti-CCR5 (1:1000; Santa Cruz Biotechnology) antibodies overnight at 4°C. After washing thrice with phosphate-buffered saline (PBS), the cells were treated with goat anti-mouse IgG FITC (1:1000; Abcam) and goat anti-rabbit IgG rhodamine (1:1000; Millipore). Finally, the cells were stained with 1× DAPI (Thermo) and visualized using confocal microscopy (LSM 700; Carl Zeiss). For immunoblotting, J774 cells were homogenized in a lysis buffer and centrifuged (12000 g at 4°C) for 15 minutes.

Equal amounts of protein (30 µg) from the supernatants were separated on a 10% acrylamide gel and electro-transferred onto nitrocellulose membranes. The membranes were incubated with mouse anti-CCR4 (1:2000; Santa Cruz Biotechnology), rabbit anti-CCR5 (1:2000; Santa Cruz Biotechnology), and rabbit anti-β-actin (1:2000; Santa Cruz Biotechnology) antibodies overnight at 4°C after blocking. The membranes were then incubated with goat anti-mouse and rabbit horse radish peroxidase (1:2000; Thermo). The blots were rinsed, and the protein bands were visualized and photographed using the ECL™ Western Blotting Analysis System (GE Healthcare) and the LAS 4000 mini (Fujifilm), respectively.

### *In Vitro* and *In Vivo* LPS and Antagonist Treatments

For the *in vitro* LPS treatment, BV2 and J774 cells were treated with LPS (10 ng/ml; Sigma) for 24 hours to induce inflammatory conditions. For the *in vivo* LPS treatment, inflammation was induced using intracerebroventricular injections for 30 minutes. Briefly, anesthetized mice were placed in a stereotaxic frame and LPS [5 ng in 5 µl PBS or sham (5 µl PBS)] was injected into the right lateral cerebral ventricle (stereotaxic coordinates: 1 mm caudal to bregma, 1.3 mm lateral to sagittal suture, and 2 mm in depth).

For antagonist treatment, J774 was treated with CAS864289-85-0 (CAS) [IC50 = 39 nM; CCR4 antagonist] and Maraviroc (Marav) [IC50 = 12.5 nM; CCR5 antagonist] for 1 hour and then seeded in the upper compartment of the transwell system. Mice were treated with CAS (5 mg) and Marav (10 mg) *via* an intraperitoneal injection 24 hours after LPS treatment.

### Intravital Imaging

Mice were injected intraperitoneally with a mixture of Zoletil (100 mg/kg) and xylazine (Rompun, 10 mg/kg) for anesthesia during imaging. Cranial window surgery and imaging were performed as described previously [15]. Body temperature was maintained at 36.5-37.5°C with a heating pad system (Live Cell Instrument). For visualization of brain vessels, mice were injected with Texas red-conjugated dextran 70-kDa (Sigma) or CF^®^405M-conjugated wheat germ agglutinin (Biotium) *via* the retro-orbital sinus. Imaging was then performed using a two-photon microscope (LSM7MP; Carl Zeiss). Brain images were obtained with light wavelength at 820 nm or 880 nm for green, red, and blue. Imaging was performed using a resolution of 512 × 512 pixels with a 20× water-immersion objective lens. The imaging depth was 40-50 µm from the cerebral cortex surface with a z-stacking system of step sizes every 1 µm. Imaris (Bitplane) was used for 3D imaging data analysis.

### Immunohistology, Cell Count, and RFP Intensity

After LPS treatment, brain sections (15 µm thick) were permeabilized with citrate buffer and blocked with 3% goat serum in PBS for 1 hour at room temperature. ProLong™ Diamond Antifade Mountant with DAPI (Invitrogen) was used for sample mounting and nuclear staining. A confocal microscope (LSM 700; Carl Zeiss) was used to observe the stained sections.

Cell counts and intensity of RFP expression were assessed using perfusion with 4% PFA. Each extracted whole brain was placed on a black paper to prevent autofluorescence in the background. RFP-positive cell counts were analyzed using ZEN blue desk top image analysis program (Carl Zeiss). All setting areas were analyzed by setting up ID and Area, and all groups were analyzed by “Batch” setting for constant analysis value. RFP intensity of whole brain of CCR2::RFP mice was measured using IVIS Spectrum CT (PerkinElmer).

### CLARITY Imaging

To create transparent tissues, extracted brain was placed in 20 ml hydrogel (40% acrylamide, 2% bis-acrylamide, 10% wt VA-044 initiator, 40 ml PBS, 100 ml 16% PFA, and 210 ml dH_2_O) for 2 days. To establish the bond between the acrylic amides, the thermal addition process is passed by desiccation chamber in a fume hood. Hydrogel-soaked brain sample was incubated 37°C for 3 hours on the rotator in a fume hood. Thereafter, the sample was sliced into 2-mm thick sections by vibratome and incubated in a clearing solution (123.66 g boric acid, 400 g sodium dodecyl sulphate, dH_2_O, and pH 8.5) at 37°C for 7 days. The clearing solution was replaced once every 3 days. The brain was then enclosed between two cover glass-bottom petri dishes. Brain imaging was performed using confocal laser scanning microscopy (LSM 5 EXCITER; Carl Zeiss) at excitation wavelengths of 647 nm. Finally, images were analyzed using Imaris software (Bitplane).

### Statistical Analysis

All experiments were randomized, and all analyses were carried out by investigators blinded to experimental conditions. Statistical analyses were performed using *t* test (Systat Software, Inc). Values were presented as the mean ± SE. Significant differences between two groups were analyzed using an unpaired *t* test, with P<0.05 considered statistically significant. Multiple comparisons were performed using one-way analysis of variance followed by Bonferroni’s *post hoc* test.

## Results

### BV2 Modulated J774 Migration in Inflammatory Conditions and Migration-Related Chemokines Are Increased in BV2 CML

**Figure 1:**
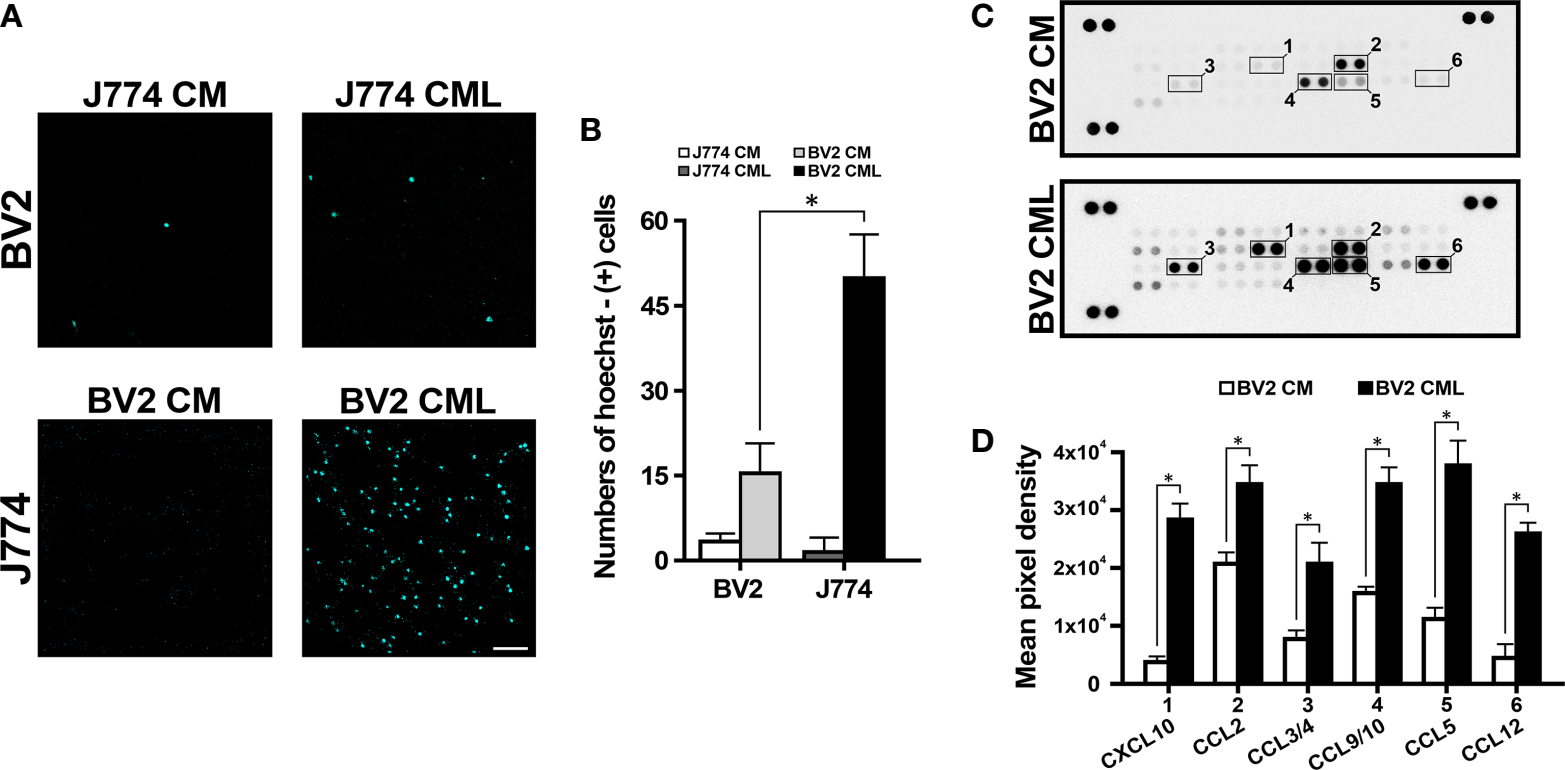
J774 migration induced by BV2. **(A)** The lower compartment of transwell system showed J774 migration. **(B)** In CML, the number of migrating J774 cells is more than three times higher than the number of migrating BV2 cells. **(C)** Representative chemiluminescence images of the proteome profiler array of CM and CML of BV2. The dotted line box indicates the selected candidate chemokines. **(D)** The quantification analysis shows the expression of CXCL10, CCL2, CCL3/4, CCL9/10, CCL5 and CCL12 in BV2 CML (n=3/group, *P<0.01, scale bar=10 µm).

To investigate the role of BV2 cells in J774 cell migration and vice versa, the CM and CML of each cell line were collected and cross-treated with each cell using a transwell system. The CM of BV2 and J774 did not affect migration of each cell. However, the CML of BV2 and J774 induced migration (**Figure 1A**). The number of Hoechst-positive J774 cells on BV2 CML was 3.5-fold higher than for Hoechst-positive BV2 cells on J774 CML (**Figure 1B**). Proteome profiler chemokine assay to screen for chemokine-associated migration in BV2 CML (**Figure 1C**) showed increased expression of several chemokines in BV2 CML. Expression levels of six chemokines, namely, CXCL10, CCL2, CCL3/4, CCL9/10, CCL5, and CCL12 were significantly higher in BV2 CML than in BV2 CM (**Figure 1D**). A search of major receptors related to the discovered chemokines identified CCR4 and CCR5 (**Table 1**).

**Table 1:** Information about the type and functions of expressed chemokines in BV2 cells.

Structure of chemokine classes	Function of Chemokines	Chemokine ligands	Chemokine receptors
**C-C(β)**	**Regulating macrophage recruitment**	**CCL2**	**CCR2, CCR4**
**C-C(β)**	**Recruitment and activation of polymorphonuclear leukocytes**	**CCL3**	**CCR2, CCR4**
**C-C(β)**	**Chemoattractant for natural killer cells, monocytes and a variety of other immune cells**	**CCL4**	**CCR5**
**C-C(β)**	**Chemotactic for T cells, eosinophils, and basophils**	**CCL5**	**CCR3, CCR5**
**C-C(β)**	**Induced by proinflammatory cytokines like IL-1 and TNF**	**CCL9,10**	**CCR5**
**C-C(β)**	**Function as anti-inflammatory mediators**	**CCL12**	**CCR4**
**C-X-C(α)**	**Pro-inflammatory cytokine**	**CXCL10**	**CXCR3**

Underline words mean ‘Selected target chemokine’.

### CCR4 and CCR5 Were Expressed in J774

**Figure 2:**
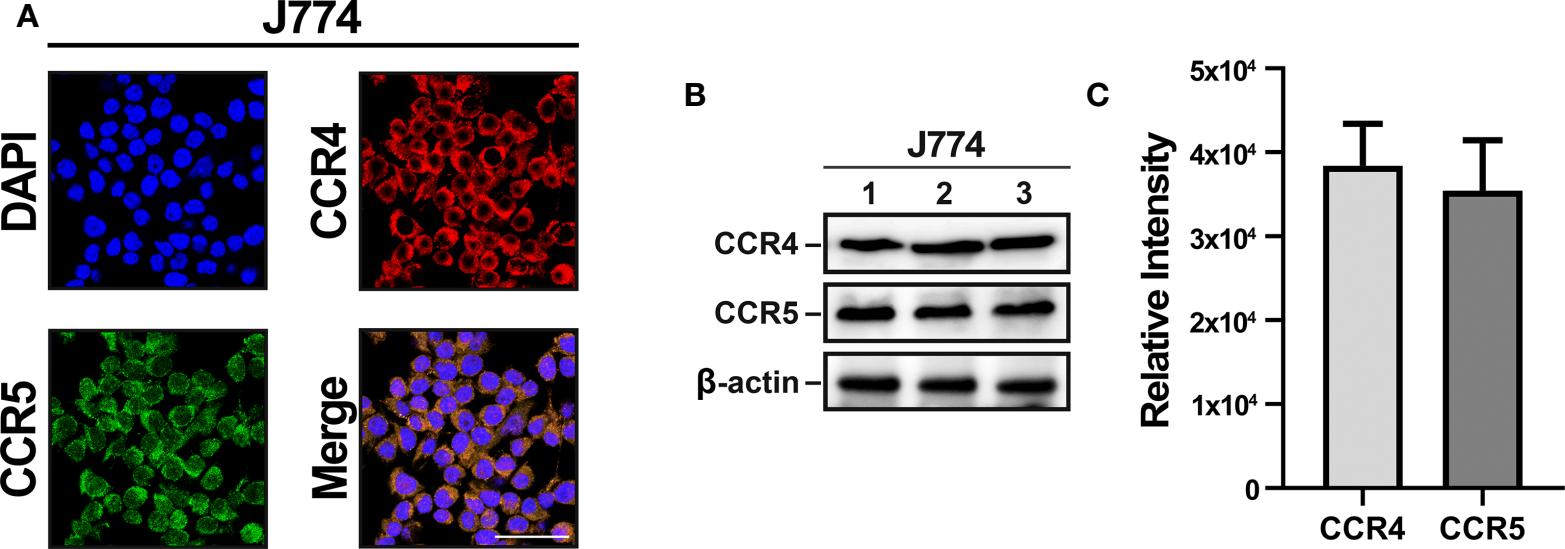
CCR4 and CCR5 expression in J774 cells. **(A)** Confocal images show similar levels of CCR4 and CCR5 expression in the J774 membrane. **(B)** Immunoblotting detected by HRP oxidation also indicates CCR4 and CCR5 expression. **(C)** Quantitative results of CCR4 and CCR5 through Image J. (n=3/group, scale bar=25 µm).

CCR4 and CCR5 were accordingly selected as target receptors, and immunocytochemistry and immunoblotting of J774 were performed to confirm CCR4 and CCR5 expression. Immunocytochemistry showed that CCR4 and CCR5 were expressed and merged in J774 (**Figure 2A**). Immunoblotting also indicated positive CCR4 and CCR5 expressions and there was no statistical change in them (**Figures 2B, C**). Collectively, these data indicated that monocyte-derived macrophages with CCR4 and CCR5 migrate into parenchymal tissues during the neuroinflammatory response.

### CCR4 and CCR5 Inhibited and Interrupted J774 Migration

**Figure 3:**
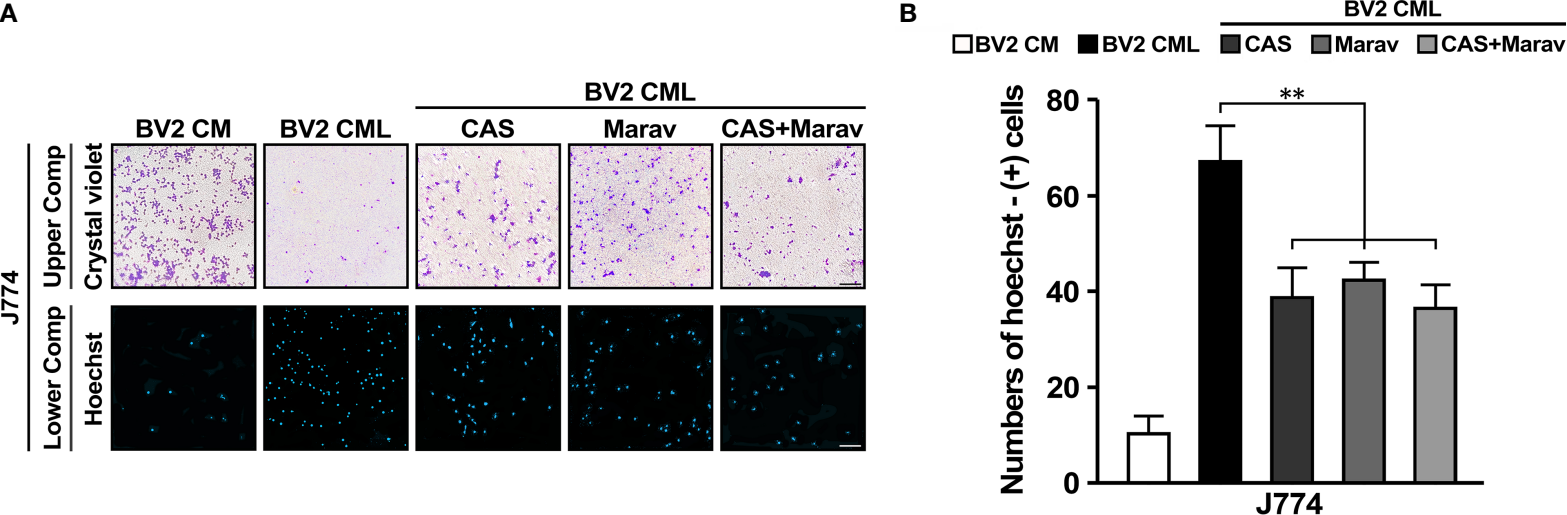
CCR4 and CCR5 antagonists interrupt migration in BV2 CML. **(A)** J774 cells in the upper compartment of the transwell system are identified using crystal violet staining and using Hoechst staining in the lower compartment. **(B)** The number of J774 cells migrating to the lower compartment is confirmed by quantitative analysis. CAS, Marav, and CAS+Marav groups in BV2 CML show significantly lower J774 migration than did not treat antagonist group in BV2 CML (n=3/group, **P<0.05, scale bar=10 µm).

To determine the functional significance of CCR4 and CCR5, J774 treated with CAS and Marav cultured CML of BV2 and confirmed J774 migration using transwell system again. A high rate of Hoechst-positive J774 cells were observed in the lower compartment of CML. In addition, the Hoechst positivity rate was lower in J774 cells treated with CAS and Marav than in untreated cells. Similar findings were observed in J774 cells treated with both CAS and Marav (CAS+Marav). Meanwhile, crystal violet positivity in the upper compartment was increased in CAS, Marav, and CAS+Marav treatment groups than in the non-treatment group (**Figure 3A**). The number of Hoechst-positive J774 cells was lower by approximately 40-45% in the CAS and Marav groups than in the LPS group. Further, the number of Hoechst-positive cells was also lower by 38-40% in the CAS+Marav group than in the LPS group (**Figure 3B**). However, there was no significant difference in Hoechst-positive J774 cells among the CAS+Marav, CAS, and Marav groups.

### Inhibition of CCR4 and CCR5 Suppressed Inflammation-Induced Migration of Monocyte-Derived Macrophage to Brain Parenchymal Tissue in the Vivo Model

**Figure 4:**
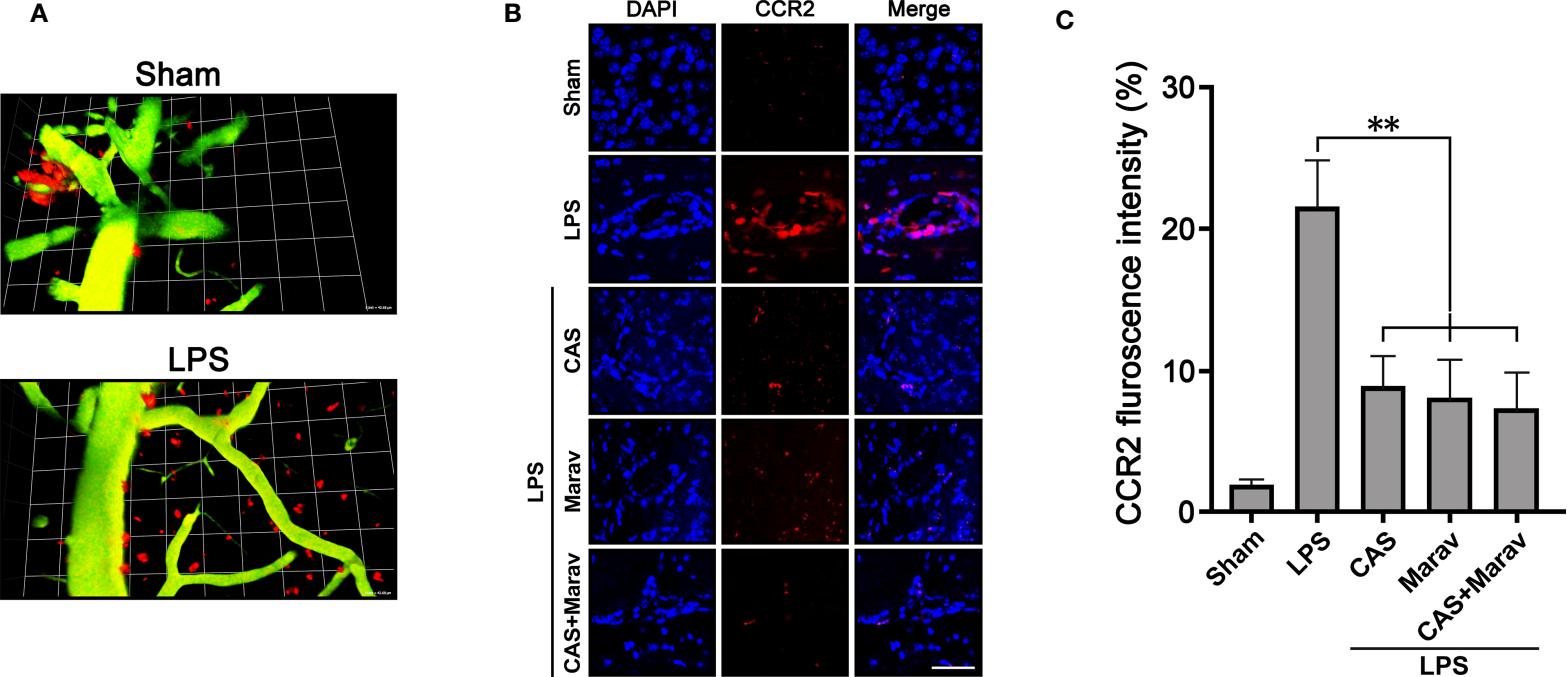
Animal model of inflammation showing migration of CCR2::RFP cells into brain parenchymal tissue. **(A)** Intravital imaging confirms that CCR2::RFP cells emerge from a brain vessel in the LPS treatment group. **(B)** CCR2::RFP cells are increased in brain tissue injected with LPS. The inflamed brain tissue treated with CAS, Marav, and CAS+Marav have decreased CCR2::RFP cells. **(C)** Graphs showing that the number of CCR2::RFP positive cells are decreased in the CAS, Marav, and CAS+Marav groups (sham intravital imaging group, n=1; LPS intravital and immunohistology imaging group, n=3; **P<0.05, scale bar=25 µm).

To investigate the migration of monocyte-derived macrophage into brain parenchymal tissue under inflammatory conditions, we used CCR2::RFP mice to observe the monocyte-derived macrophage migration at the inflammation site. Two-photon intravital imaging showed CCR2::RFP cells were migrating out of dextran-stained brain blood vessels in the LPS treatment group (**Figure 4A**). Immunohistology indicated CAS and Marav suppressed CCR2::RFP cells in the LPS-treated parenchymal brain (**Figure 4B**). The number of CCR2::RFP-positive cells was lower by approximately 60-70% in the CAS, Marav, and CAS+Marav groups than in the LPS group. Among the mice treated with LPS, the CAS+Marav group showed a similar tendency *in vitro* (**Figure 4C**).

**Figure 5:**
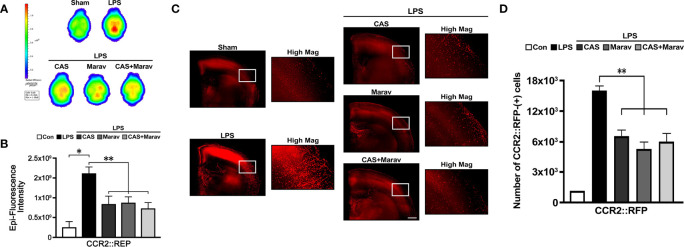
CCR2::RFP cell migration in neuroinflammation condition. **(A)** IVIS spectrum showing the ex vivo brain image with RFP after LPS injection. **(B)** The fluorescence intensity CCR2::RFP cells is lower in the CAS, Marav, and CAS+Marav groups than in the nontreatment group. **(C)** CLARITY images and also show a lower intensity of CCR2::RFP cells in the CAS, Marav, and CAS+Marav groups than in the nontreatment group. High magnification images (Hig Mag) also showed the same the condition. **(D)** Graphs showing the number of CCR2::RFP-positive cells are decreased in the CAS, Marav, and CAS+Marav groups (sham clarity group, n=1; Experimental CLARITY group, n=3; IVIS group, n=3; *P<0.01, **P<0.05, scale bar=500 µm).

Given that immunohistology only identifies a limited area, the efficacy of CCR4 and CCR5 inhibitors on the whole brain cannot be determined. Therefore, we clarified the effect on a wider brain area and performed IVIS Spectrum and CLARITY imaging. The results of IVIS Spectrum showed stronger RFP intensity in the LPS group than in the CAS, Marav, and CAS+Marav groups (**Figure 5A**), with RFP intensity being approximately 50-60% lower in the CAS, Marav, and CAS+Marav groups (**Figure 5B**). Furthermore, CLARIITY imaging showed lower RFP positive cells in the LPS group than in the CAS, Marav, and CAS+Marav groups in parenchymal tissue of mice brain (**Figure 5C**). The number of CCR2::RFP-positive cells in the CAS, Marav, and CAS+Marav groups were lower by approximately 55-65% than that in the LPS group (**Figure 5D**). As expected, IVIS Spectrum and CLARITY imaging showed no significant difference in RFP expression between the CAS+Marav group and the CAS and Marav groups.

## Discussion

Evidence on the association between monocyte-derived macrophages and microglia in neuroinflammation that occurs in many neurological diseases, including stroke. This study observed the following findings. First, the migration of monocyte-derived macrophages during inflammation is induced by microglia. Second, CCR4 and CCR5 are monocyte-derived macrophage migration targets. Third, the inhibition of CCR4 and CCR5 halted the migration of monocyte-derived macrophages into parenchymal tissues in the neuroinflammatory response. Collectively, these results support that microglia-induced migration of monocyte-derived macrophages may aggravate the inflammatory response in the brain, and thus, inhibition of infiltration of these cells can alleviate neuroinflammation. After acute brain injury, microglia are increased and converted to an active, motile ameboid state (16, 17). Dynamic microglia are considered resident CNS macrophages. Activated microglia and macrophages are distributed throughout the entire lesion and are detectable up to 1 week after the insult (18). Activated microglia and macrophage exhibit chimeric characteristics. These cells have been shown to affect neuronal cell death and may be essential for tissue repair and wound healing (19, 20). However, prolonged brain injury also induces microglia and macrophage overactivation and the release of pro-inflammatory factors, such as cytokines and chemokine (21). In neuroinflammation of neurological disease, although the properties of microglia and blood-derived macrophages are well known, the interactions between these cells remain unclear.

We previously established that monocytes infiltrated into the parenchymal tissue of the injured brain, and these cells were converted to M2 phenotype macrophage by cytokine released from microglia, such as IL-4 and IL-13 (15). Following previous studies, in this study, we investigated the mechanism by which monocyte-derived macrophage infiltrated parenchymal brain tissue during neuroinflammatory response. In the transwell system of BV2 CML, an increased infiltration of J774 cells was found, and chemokines present in BV2 CML were analyzed. Screening of chemokines by proteome profiler assay showed that CXCL10, CCL2, CCL3/4, CCL9/10, CCL5, and CCL12 expression were higher in BV2 CML than in control. CCR4 and CCR5 were the receptors that mainly interacted with these chemokines. CCRs transduce signals through pertussis toxin-sensitive Gai G-proteins and β-arrestins, ultimately leading to cell migration, adhesion, and/or various other biological responses. In addition, it was confirmed that the migration of monocyte in the blood to the brain parenchymal tissue was reduced when the antagonists of CCR4 and CCR5 were treated in a neuroinflammation animal model induced by LPS.

Many studies suggested that CCR4 is a key player in regulating lymphocytes involved in systemic immunity (22). CCR4 was also expressed in other cell types, such as platelets, monocytes, and macrophages (23–25). CCR4-deficient mice were found to have resident macrophages with an alternatively activated phenotype (26). These mice had reduced levels of pro-inflammatory cytokines and had interrupted migration of macrophages into the peritoneal cavity (25). In ischemic stroke, the polarization of microglia/macrophage is partly dependent on CCR4, which is involved with the activation of the NF-κB pathway related with CKLF1 (27). However, studies on the role of CCR4 in monocyte/macrophage migration are rare. To our best knowledge, our study is the first to report that CCR4 induces migration of monocyte-derived macrophage; accordingly, a CCR4 antagonist inhibited inflammation.

CCR5 is a member of the G protein-coupled receptor, a seven-transmembrane receptor family that regulate trafficking and function of memory/effector T-lymphocytes, macrophages, and immature dendritic cells (28, 29). CCR5 is known to be involved in the recruitment of monocyte-derived macrophages to the injured central nervous system (30). CCR5 and their ligands are important in the interaction among parenchymal liver cells, Kupffer cells, hepatic stellate cells, and infiltrating immune cells during liver inflammation (31). Pharmacological inhibition and genetic deficiency of CCR5 lead to immune cell inactivation and reduced liver fibrosis in an animal hepatic fibrosis model (32). A recent study showed that CCR5-deficient mice have increased systemic inflammatory response and mortality in polymicrobial sepsis models (33). However, some studies also showed that CCR5 is not involved in the migration of monocyte from the bone marrow (34).

In conclusion, although the relationship between microglia and monocyte-derived macrophages remains unknown, the current study findings indicate that CCR4 and CCR5 may play an important role in the interaction between microglia and monocyte-derived macrophage in necroinflammation conditions inducted by microglial activation. Cytokines released by microglia induce the infiltration of monocyte-derived macrophages into brain parenchymal tissue after inflammation; accordingly, inhibition of receptors related with these cytokines led to opposite observations. Targeting CCR4 and CCR5, which induce the infiltration of monocyte-derived macrophages, could alleviate neuroinflammation conditions caused by microglial activation. Therefore, future studies should further address the optimal timing and dosing of CCR4 and CCR5 inhibitors for clinical application.

## Data Availability Statement

The raw data supporting the conclusions of this article will be made available by the authors, without undue reservation.

## Ethics Statement

The animal study was reviewed and approved by The Institutional Animal Care and Use Committee of Yonsei University Health System and according to the National Institutes of Health guidelines.

## Author Contributions

JEL contributed to study conception and design. Material preparation, data collection, and analysis were performed by JYK, JK, and MH. The first draft of manuscript was written by JYK. JK and RK commented on previous version of the manuscript. All authors contributed to the article and approved the submitted version.

## Funding

This study was supported by the National Research Foundation of Korea (NRF) grant funded by the Ministry of Science, ICT, and Future Planning (NRF-2016M3C7A1905098 to JEL), the NRF grant funded by the Korea government (MSIT) (NRF-2021R1A2C2008034 to JEL), and NRF funded by the Ministry of Education (NRF-2020R1I1A1A01064803 to JYK).

## Conflict of Interest

The authors declare that the research was conducted in the absence of any commercial or financial relationships that could be construed as a potential conflict of interest.

## Publisher’s Note

All claims expressed in this article are solely those of the authors and do not necessarily represent those of their affiliated organizations, or those of the publisher, the editors and the reviewers. Any product that may be evaluated in this article, or claim that may be made by its manufacturer, is not guaranteed or endorsed by the publisher.
